# Metropolitan Hospital Sunday Fund

**Published:** 1909-12-04

**Authors:** 


### METROPOLITAN HOSPITAL SUNDAY FUND.

A meeting of the Council of the Metropolitan Hospital-Sunday Fund was held at the Mansion House on Tuesday, November 30, under the presidency of the Right Honourable the Lord Mayor. The Lord Mayor expressed his pleasure at meeting the Council for the first time in his Mayoralty, and said he hoped it would be by no means the last time he would have that satisfaction.

Sir John Charles Bell, Bart., moved the adoption of the report of the Council for 1909. The figures, he said, showed that the collections were about £1,000 less than in the previous year. All must admit that that was a highly gratifying state of things, considering the time through which we had passed. In fact, that difference could be accounted for by the smaller amounts in three or four very large offertories, due to changes which had taken place in those places of worship. Their gratitude was again due to the clergy of all denominations who had done so excellently well as to provide the Fund with so large a sum—nearly £40,000. He regretted to say that the hand of death had been rather heavy upon them during the last year, having removed some of the most active members. The late Dr. Rigg was one of the founders of the Fund, and had been a member of the Council since 1874. And the deaths of Sir Thomas Smith, Sir Stephen Mackenzie, and Thomas Wakley, Esq., were deplored by all. To fill the vacancies thus caused, the constituents at a meeting proposed the following : Sir George Truscott (late Lord Mayor), Mr. John H. Morgan, C.B., F.R.C.S., Rev. Morris Joseph, Rev. D. J. Dollar, all of whom had consented to stand if elected. Sir Frederick Treves and Mr. Alfred Willett had retired from the Distribution Committee, and it had been recommended that their places should be taken by Mr. J. H. Makins and Mr. E. Parker Young, M.R.C.S., who also had agreed to act if chosen. The adoption of the report was seconded by the Rev. Charles Brown, and carried unanimously.

Hospital Sunday was appointed as June 12 next year.

Sir William Church proposed that the valuable and long-continued work on the Distribution Committee rendered by Mr. Willett should be recognised by an official letter of thanks being sent to him, which Mr. F. H. Norman seconded, and the resolution was carried without dissent.

The Secretary reminded the Council that Mr. Lionel Lewis had given notice of his intention to move, at the annual meeting to be held on December 17, that the law of the constitution be altered so that the Distribution Committee might be elected by the constituents. Mr. Lewis was within his right to move such a resolution; but it would alter radically the procedure which had been followed for the last 37 years.

Mr. Hale said it would be a good thing to refer the proposal to the Distribution Committee.

Archdeacon Sinclair said the matter had already come before the Committee, who had dealt with it. He considered it would be a most unhappy innovation, for the reasons which the Secretary had stated. Many of those now serving had done so for many years, and had grown old in it, so that they knew what was necessary for its prosperous maintenance. To have a chance committee selected by the annual meeting, which was really an adventitious gathering, would be almost impossible. He had no doubt that it would be rejected.

Sir Henry Burdett said the suggestion just made with reference to the Distribution Committee was really like referring the matter back to themselves, as it affected them. The policy of the Distribution Committee was that it should be absolutely impartial and neutral; but if they were to throw open the election of the members of it to a great meeting in the Egyptian Hall once a year he was certain it would undermine the foundation-stone which made for the popularity of the Fund. That foundation-stone was that during all those years the gentlemen who had been selected for their impartiality, from the fact that they were not directly connected with the management of any participating hospital, had so managed matters that no award which they had made had been seriously challenged in the whole history of the Fund. If anyone wanted a reason why it would be unwise to interfere with the present system, no better one could be found than in the record of the work of the Distribution Committee.

The meeting was concluded by a vote of thanks to the Lord Mayor.

